# Remarks on the Nature and Treatment of Intermittents, as They Occurred at Hampstead in the Spring of 1781

**Published:** 1781-10

**Authors:** 


II. *Remarks on the nature and treatment of Intermittents, as they occurred at Hampſtead in the ſpring of* 1781. *Extracted from a letter addreſſed to the Society by Mr.* Thomas Hayes, *ſurgeon at Hampſtead. Read* October 8, 1781.

ABOUT the beginning of January 1781, the meaſles were very rife in Hampſtead and its environs. In the greater number of caſes they ended in very troubleſome intermittents, that gave way only to bleeding, and an antiphlogiſtic treatment; the bark, when adminiſtered, having been generally found to do harm.

Theſe meaſles continued to appear in different parts of the neighbourhood till the month of June, when the nature of the diſeaſe ſeemed to be materially changed; for after a purge or two the bark generally ſucceeded, while bleeding had uniformly a bad effect.

In the month of February a great number of children and adults were attacked with all the ſymptoms of common intermittents, but the liver ſeemed to be more than ordinarily affected. Such as were athletic were uſually ſeized with uncommon violence, having a ſtrong pulſe, accompanied with delirium, erratic pains in the region of the liver, cheſt, ſhoulders, &c.

Great marks of plethora appeared in the greater number of the patients, and evident ſymptoms of inflammatory diatheſis pointed out bleeding as the firſt remedy to be employed. I had generally obſerved, that vernal agues were relieved by it, whilſt it almoſt always did harm in autumnal ones; but now, much to my ſurprize, it very ſeldom was attended with advantage; and a ſecond bleeding evidently prolonged the the difeafe, as I frequently remarked in thofe patients who had applied to country bleeders and repeated that operation before they had other affiftance. The blood taken away was not very fizy; but the craffamentum was blacker than ufual, and the ferum more yellow and vifcid than common. A vomit was always given, and fometimes repeated; the faline mixture, joined with emetic tartar, and a purge were interpofed till the intermiffions were perfect and diftinct, that the bark might be given with fafety and benefit, a caution always to be attended to before it is adminiftered. Notwithftanding all this care, double, nay, treble the ufual quantity of bark, given in a variety of forms, did not remove the difeafe, or prevent a return of it: myrrh, fnake-root, &c. were joined to it, but with no better fuccefs. If it ftopped for a week or two, it returned, though the bark was continued.

Finding that thefe difficulties were pretty general, I adopted the following mode of treatment, which, though it has not the claim of novelty to recommend it, will perhaps not be deemed unworthy the attention of the Society on account of the fuccefs that attended it. Several of my friends to whom I recommended it, found it equally efficacious.

I began

I began by giving an emetic of ipecacoanha and emetic tartar in the evening, which was repeated if neceſſary a few days after. If the diſeaſe was accompanied with great marks of plethora, or inflammatory diatheſis, I took away a few ounces of blood; but the number of patients who required this evacuation was very ſmall, and I do not recollect a ſingle caſe in which I had occaſion to repeat it. After the emetic I preſcribed half a drachm of ſal Polychreſt to be taken night and morning for a week or ten days, and if the fever was high, the patients took three or four ſpoonfuls of a ſaline mixture with calx of antimony ſeveral times a day. The fits were ſuffered to go on for ſeveral days. In ſome the fever aſſumed the type of a quotidian, in others that of a tertian, or double tertian. By purſuing this method the fits became gradually leſs, and the general health of the patient got better, and often well without any other remedy. Several had recourſe to a decoction of bark with elixir of vitriol, and the uſe of a chalybeate ſpring, of which we have a remarkable good one at this place, and obtained perfect health.

But ſome patients, who had neglected themſelves in the beginning, or taken the bark prematurely, maturely, got into a train of ill health from obſtructed viſcera, &c. and were obliged to have recourſe to more active medicines, of which none ſucceeded ſo well as crude quickſilver carefully triturated with mucilage of gum arabic, and given in doſes of ten, fifteen, or twenty grains night and morning, the patient drinking after each doſe a quarter of a pint of a ſtrong decoction of Taraxacum, in which was diſſolved a drachm of ſoluble tartar. The doſe of the latter was diminiſhed if the medicine purged the patient; this method was purſued for a week or ten days, after which the following pills generally completed a cure in a ſhort time:

℞ Aloes. ſocot.

Gum. ammoniac.

—— myrrh.

Calc. antimon. illot.

Sapon. venet. āā ℥j.

Ol. chamæm. chym. gutt. xx.

Extract. gentian. q. ſ. ut fiat maſſa, ex cujus ſingulis drachmis formentur pilulæ xij.

Of theſe pills the patient took two, three, or four twice or three times a day, waſhing down each doſe with four ounces of an infuſion of centaury or chamomile flowers.

*Hampſtead,*
Sept. 8th, 1781.

SECTION

